# Antioxidative Activities and Active Compounds of Extracts from *Catalpa* Plant Leaves

**DOI:** 10.1155/2014/857982

**Published:** 2014-11-05

**Authors:** Hongyu Xu, Gege Hu, Juane Dong, Qin Wei, Hongbo Shao, Ming Lei

**Affiliations:** ^1^College of Forestry, Northwest A&F University, Yangling, Shaanxi 712100, China; ^2^College of Life Sciences, Northwest A&F University, Yangling, Shaanxi 712100, China; ^3^Key Laboratory of Coastal Biology & Bioresources Utilization, Yantai Institute of Coastal Zone Research (YIC), Chinese Academy of Sciences (CAS), Yantai 264003, China; ^4^Jiangsu Academy of Agricultural Sciences, Nanjing 210014, China

## Abstract

In order to screen the *Catalpa* plant with high antioxidant activity and confirm the corresponding active fractions from *Catalpa ovata* G. Don, *C. fargesii* Bur., and *C. bungei* C. A. Mey., total flavonoid contents and antioxidant activities of the extracts/fractions of *Catalpa* plant leaves were determined. The determined total flavonoid content and antioxidant activity were used as assessment criteria. Those compounds with antioxidant activity were isolated with silica gel column chromatography and ODS column chromatography. Our results showed that the total flavonoid content in *C. bungei* C. A. Mey. (30.07 mg/g*·*DW) was the highest, followed by those in *C. fargesii* Bur. (25.55 mg/g*·*DW) and *C. ovata* G. Don (24.96 mg/g*·*DW). According to the determination results of total flavonoid content and antioxidant activity in 3 clones of leaves of *C. bungei* C. A. Mey., the total flavonoid content and antioxidant activity in crude extracts from *C. bungei* C. A. Mey. 6 (CA6) leaves were the highest. Moreover, the results showed that the total flavonoid content and antioxidant activities of ethyl acetate (EA) fraction in ethanol crude extracts in CA6 leaves were the highest, followed by *n*-butanol, petroleum ether (PE), and water fractions. Two flavonoid compounds with antioxidant activity were firstly isolated based on EA fraction. The two compounds were luteolin (**1**) and apigenin (**2**), respectively.

## 1. Introduction

The damage effect of free radicals on organisms can be inhibited by antioxidants and the inhibition effect has been recognized as a potential therapeutic way for the prevention and treatment of related diseases and become a hotspot in the field of preventive medicine [1]. The advantages of synthetic antioxidants include high efficiency and low cost. However, synthetic antioxidants also induced many security problems like the negative impact on enzyme systems in human beings [2]. On the contrary, natural antioxidants present high efficacy and small disadvantages. In this sense, compared with the synthetic antioxidants, it is necessary for the food industry to develop new effective and safe antioxidants from plants.

The* Catalpa* plants of Bignoniaceae family have about 13 species in the world and are mainly distributed in America and East Asia. Five species and 1 variant of* Catalpa* plants were introduced into China:* C. ovata* G. Don,* C. bungei* C. A. Mey.,* C. fargesii* Bur., variant of* C. tibetica* Forrest,* C. speciosa* Ward., and* C. fargesii* f.* duclouxii* Dode, respectively. Previous phytochemical investigations indicated that iridoids and naphthoquinones were the main constituents of* Catalpa* plants [3–8];* C. ovata* G. Don have showed antifungal activity [9, 10], anti-inflammatory activity [11–15], antitumor activity [7, 16–18], antioxidant activities [4, 19], and so on. However, the bioactivity of other* Catalpa* plants was seldom reported.

In this paper, the scavenging activity of DPPH radical and hydroxyl radicals and the reducing power of crude extracts from the leaves of* C. ovata* G. Don,* C*.* fargesii* Bur., and* C*.* bungei* C. A. Mey. were analyzed, in order to screen the* Catalpa* plant or clones with the highest antioxidant activity. Furthermore, the antioxidant activities of different polar fractions (ethyl acetate (EA),* n*-butanol, petroleum ether (PE) fraction, and water fraction) from ethanol crude extracts of* C. bungei* C. A. Mey. 6 (CA6) leaves were analyzed so as to obtain the highest antioxidant activity group. Moreover, further separation and purification against the highest antioxidant activities groups to identify the structure of compounds with antioxidant activities were performed. This study may lay a foundation of breeding* Catalpa* plant with strong antioxidant activity for the development of natural antioxidants. At the same time, the study may also provide a theoretical basis for searching natural antioxidant active ingredients from nature as resources of development of new drugs and health care.

## 2. Materials and Methods

### 2.1. General

Melting points were determined with a digital melting-point apparatus and were uncorrected. Electrospray ion trap mass spectrometry (ESI-MS) was carried out with Bruker ESI-TRAP Esquire 6000 plus mass spectrometry instrument. Nuclear magnetic resonance spectra (NMR) were recorded on a Bruker Avance III 500 MHz instrument in DMSO-*d6* with tetramethylsilane (TMS) as the internal standard. Analytical thin-layer chromatography (TLC) was performed with silica gel plates and silica gel 60 GF_254_ (Qingdao Haiyang Chemical Co., Ltd.).

### 2.2. Plant Material and Chemicals

Leaves of* C*.* ovata* G. Don and* C*.* fargesii* Bur. were collected from Yangling of Shaanxi Province, China (East longitude 108° 08′, latitude 34° 27′, 440 m above sea level). Leaves of* C*.* bungei* C. A. Mey. were acquired from Tianshui in Gansu Province, China (East longitude 105° 41′, latitude 34° 14′, 1131 m above sea level). Chosen three plant materials were widely distributed in China and their leaves were collected in November 2013. A certain amount of healthy leaves with similar foliar age was collected. Leaves were heated for 20 min in an oven at 90°C immediately after collection for deactivation of enzymes and then dried at 60°C. After crushing, the leave samples were filtered with a 20-mesh sieve and then stored.

1,1-Diphenyl-2-picrylhydrazyl (DPPH), Folin-Ciocalteu reagents, and rutin (>99%) were obtained from Sigma (St. Louis, USA). 2-Deoxy-D-ribose was obtained from Aladdin (Shanghai, China). Butylated hydroxyanisole (BHA) was analytical reagents obtained from Bodi (Tianjin, China). All the reagents and solvents were of reagent grade or purified according to standard methods before use.

### 2.3. Determination of Total Flavonoids Contents

Total flavonoid content was determined according to the method reported by Zhishen et al. [20] with some modifications. Each sample (1 mL) was added into a 10 mL test tube, and then 3 mL methanol and 5% NaNO_2 _(0.3 mL) were added after 6 min. Then, 0.3 mL 10% Al(NO_3_)_3 _was added. After 6 min, 4 mL 1.0 M NaOH and 0.4 mL methanol were added, and the mixture stood alone at room temperature (RT) for 15 minutes. At last, it was measured against methanol as a blank at 510 nm. With the solution of rutin (0.50–4.00 mg/mL) as the standard, a calibration curve was plotted to calculate the content of total flavonoids.

### 2.4. DPPH Radical Scavenging Activity Assay

The DPPH radical scavenging activity assay was carried out according to the method reported by Brand-Williams et al. [21] with some modifications. Each sample (1 mL) was mixed with 3 mL 0.2 mM DPPH ethanol solutions. Then, 4 mL ethanol was added and incubated at RT in dark for 30 min. The absorbance was measured with spectrophotometer at 517 nm. The DPPH scavenging activity of each sample was calculated according to (1). The concentration of sample or standard antioxidant for the 50% DPPH scavenging (IC_50_) was also calculated. The value of IC_50 _was opposite to the DPPH radical scavenging activity of samples. The lower IC_50_ value indicates the higher DPPH radical scavenging activity. Consider

(1) $$\begin{array}{c} \text{scavenging}\;\text{activity}\left(\%\right)=\frac{A_{\text{c}}-A_{\text{s}}}{A_{\text{c}}}\times 100. \end{array}$$

Note that *A*_c_ = the absorbance of the control; *A*_s_ = the absorbance of the sample solution.

### 2.5. Hydroxyl Radical Scavenging Activity Assay

Hydroxyl radical scavenging activity assay was performed according to the previously reported method [22]. Each sample (200 *μ*L) was mixed with the mixture of 100 *μ*L FeSO_4 _(10 mM), 100 *μ*L EDTA (10 mM), 500 *μ*L a-deoxyribose (10 mM), and 900 *μ*L sodium phosphate buffer (0.1 M, pH 7.4). Then, 200 *μ*L H_2_O_2_ (10 mM) was added and incubated for 1 h at 37°C. The reaction was terminated through the addition of 1.0 mL 2.8% TCA (m/v) and 1.0 mL 1.0% TBA (m/v) and incubated in boiling water for 15 min. After cooling to RT and centrifugation at 3000 r/min (TGL-16, China) for 5 min, the absorbance of the supernatant was measured at 532 nm. The hydroxyl radical scavenging activity of each sample was calculated according to (2). The concentration of sample or standard antioxidant for the 50% hydroxyl radical scavenging (IC_50_) was also calculated. Consider

(2) $$\begin{array}{c} \text{scavenging}\;\text{activity}\left(\%\right)=\frac{A_{\text{c}}-A_{\text{s}}}{A_{\text{c}}}\times 100. \end{array}$$

Note that *A*_c_ = the absorbance of the control; *A*_s_ = the absorbance of the sample solution.

### 2.6. Reducing Power Assay

Reducing power assay was carried out according to the method reported by Oyaizu [23]. Each sample (0.5 mL) was mixed with 0.5 mL sodium phosphate buffer (0.2 M, pH 6.6) and 0.5 mL 1% potassium ferricyanide and incubated for 20 min at 50°C. Then, the mixture was cooled rapidly and 0.5 mL 10% TCA was added. After mixing and centrifugation at 3000 r/min for 20 min, 1 mL supernatant was mixed with 1.0 mL distilled water and 0.2 mL 0.1% ferric chloride and then incubated for 10 min. The absorbance was detected at 700 nm. The reducing power of samples was indicated by the percentage of the activity of 0.1 mg/mL of ascorbic acid.

### 2.7. Extraction and Isolation

Each treated sample (5 g) was extracted with 50 mL 80% ethanol-water solvent (v/v) at 80°C for 1 h and extraction was performed for three times. After filtration, the filtrates of total extracts were dried at 45°C under the reduced pressure. The concentrated extracts were dissolved with distilled water to the final volume of 50 mL and stored at 4°C for analysis.

CA6 leave (100 g) sample was firstly extracted and concentrated according to the method mentioned above. Then, the concentrated extracts were dispersed in 500 mL distilled water and further extracted with 500 mL of PE EA and* n*-butanol for 5–8 times. Each fraction was dried and dissolved with distilled water at a stock concentration of 1.0 mg/mL for the determination of total flavonoid content and antioxidant activity analysis.

The extracts with the strongest antioxidant were subjected to silica gel column chromatography with the mobile phase of the mixture of chloroform : methanol (150 : 1 to 5 : 1). Through the classification by TLC, the antioxidant activity of each fraction was determined. The fraction with the higher antioxidant activity was separated and purified. The final products were compounds 1 and 2.

### 2.8. Statistical Analysis

All experiment data were expressed as mean ± SD of three experiments. Statistical analysis of ANOVA for variance and SNK test was performed with SPSS 16.0 software (SPSS Inc., Chicago, IL, USA).

## 3. Results and Discussion

### 3.1. Total Flavonoid Content and Antioxidant Activity of* Catalpa* Plants

In this study, the total flavonoid contents of crude extracts from the leaves of* C. ovata* G. Don,* C. fargesii* Bur., and* C. bungei* C. A. Mey. were determined and the antioxidant activity of these extracts by investigating the reducing power, DPPH radical scavenging activity, and hydroxyl radical scavenging activity was analyzed.

As shown in Table 1, the total flavonoid contents of the leaves from different* Catalpa* plants were significantly different (*P* < 0.05). Among these* Catalpa* plants samples, leavesof* C. bungei* C. A. Mey. showed the highest total flavonoid content (40.45 mg/g*·*DW).* C. fargesii* Bur. ranked the second position (32.52 mg/g*·*DW). The total flavonoid contents in the leaves of* C. ovata* G. Don were the lowest (29.08 mg/g*·*DW). DPPH radical scavenging activity and hydroxyl radical scavenging activity could be indicated by the value of IC_50_; the larger the value is, the lower its activity is. The crude extracts in the leaves of* C. bungei* C. A. Mey. showed the strongest reducing power, DPPH radical scavenging activity, and hydroxyl radical scavenging activity, followed by* C*.* fargesii* Bur. and* C. ovata* G. Don. The antioxidant activity of* Catalpa* plant was significantly higher than others, which may relate to the structures and contents of theantioxidative activities compounds.

### 3.2. Total Flavonoid Content and Antioxidant Activity of Clones of* C. bungei* C. A. Mey

The antioxidant activity of* C*.* bungei* C. A. Mey. (CA) is higher than those in* C*.* ovata* G. Don and* C*.* fargesii* Bur. Three clones of CA with relative strong antioxidant efficiency have been chosen as the experimental materials based on the early study (in press). And the antioxidant efficiency of 3 clones of CA (CA2, CA5, and CA6) was evaluated by the investigation of the total flavonoid content, reducing power, hydroxyl radical scavenging activity, and DPPH radical scavenging activity of crude extracts from plant leaves.

As shown in Table 2, the total flavonoid content and antioxidant activity of crude extracts from the 3 clones of the leaves of CA were significantly different (*P* < 0.05). Among the 3 clones of CA, CA6 showed the highest total flavonoids content (46.29 mg/g*·*DW), reducing power (41.61%), hydroxyl radical scavenging activity (IC_50_, 0.08 mg/mL), and DPPH radical scavenging activity (IC_50_, 0.98 mg/mL). However, CA5 showed the lowest total flavonoid content (32.17 mg/g*·*DW), reducing power (26.58%), hydroxyl radical scavenging activity (IC_50_, 0.15 mg/mL), and DPPH radical scavenging activity (IC_50_, 2.23 mg/mL).

The total flavonoid content and antioxidant activity of crude extracts from* C*.* ovate *G. Don,* C*.* fargesii* Bur., and the 3 clones of leaves of CA were significantly different. This result indicated that there was a big difference among different kinds and different clones of the* Catalpa* plant in total flavonoid content and antioxidant activities. The result was also consistent with previous reports on the difference of total flavonoid content and antioxidant activity of apples [24, 25] and pomegranate [26] resulting from fruit varieties and producing area. The accumulations of secondary metabolites in plants were mainly influenced by genetic adaptation capability and ecological environment, such as temperature, moisture, illumination, and altitude [27]. For example, plants in the high altitude area can synthesize flavonoids with the strong absorption in the ultraviolet region to reduce the UV radiation damage. Therefore, the CA6 in high altitude area, because of the strong ultraviolet radiation, has the high flavonoid content and antioxidant activity.

### 3.3. Total Flavonoid Content and Antioxidant Activity of Various Fractions of CA6

With CA6 leaves, the reducing power, hydroxyl radical scavenging activity, and DPPH radical scavenging activity of different polar fractions (PE, EA,* n*-butanol, and water fraction) extracted from ethanol crude extracts of CA6 leaves were further investigated, with BHT as positive control.

**Figure 1:**
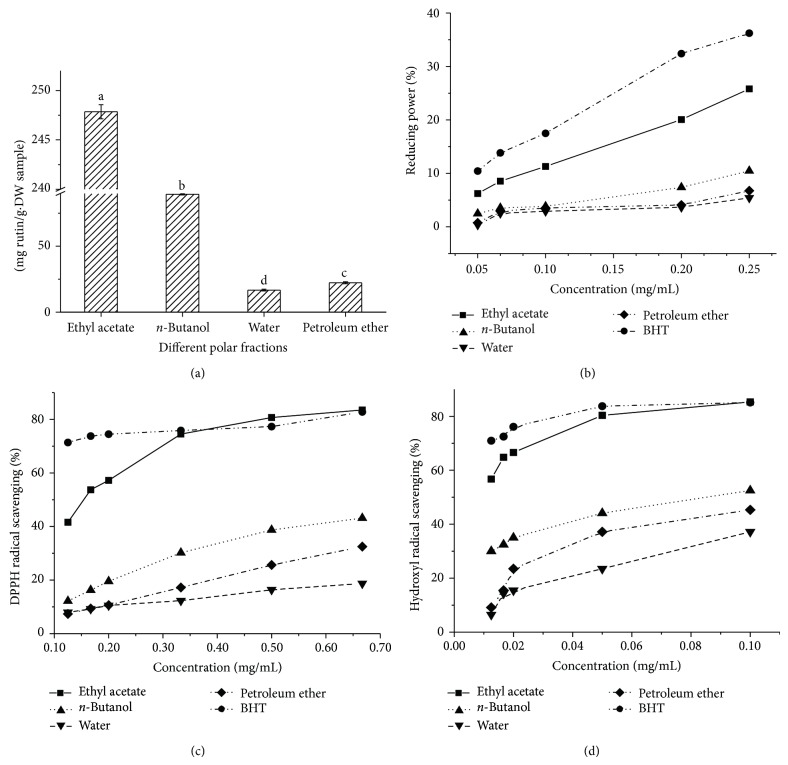
Total flavonoid content and antioxidant activity of various fractions of CA6. (a) Total flavonoid content; (b) reducing power; (c) DPPH radical scavenging activity; (d) hydroxyl radical scavenging activity.

As shown in Figure 1(a), the total flavonoid content of different polar fractions was significantly different (*P* < 0.05). From nonpolar PE to and polar water phase, all fractions contain flavonoid compounds. The total flavonoid content of EA fraction (247.85 mg/g*·*DW) was significantly higher than that of* n*-butanol (89.31 mg/g*·*DW) and PE fraction (22.24 mg/g*·*DW). The total flavonoid content of water phase (16.71 mg/g*·*DW) was significantly lower than those of others. Thus, the flavonoid compounds of CA6 leaves mainly existed in EA fraction.

As shown in Figure 1(b), all the different polar fractions of crude extracts of CA6 leaves exhibited the reducing power in a dose-dependent manner at the concentration of 0.05–0.25 mg/mL. Among these fractions, the EA fraction showed the highest reducing power, followed by the* n*-butanol fraction, PE fraction, and water residue.

As shown in Figure 1(c), the DPPH radical scavenging activity of different polar fractions showed a dose-dependent manner at the concentration of 0.13–0.67 mg/mL. Compared with the DPPH radical scavenging activity of BHT, different polar fractions displayed the significantly weaker scavenging activity at the lower concentration of 0.13–0.33 mg/mL. Only EA fraction shows similar activity to BHT at the higher concentration of 0.50–0.67 mg/mL. Among the four fractions, the EA fraction presented the highest activity at all the tested concentrations, followed by* n*-butanol fraction and PE fraction. The aqueous residue showed the lowest DPPH radical scavenging activity.

The hydroxyl radical scavenging activity of different polar fractions of crude extracts of CA6 leaves was further determined. The results showed that all the different polar fractions of crude extracts of CA6 leaves exhibited hydroxyl radical scavenging activity at the concentration of 0.01–0.10 mg/mL and showed a dose-dependent effect (Figure 1(d)). The EA fraction presented the highest hydroxyl radical scavenging activity, followed by* n*-butanol fraction, PE fraction, and aqueous residue. Compared with the hydroxyl radical scavenging activity of BHT, the EA fraction has a slightly lower hydroxyl radicals scavenging activity when its concentration was less than 0.05 mg/mL, but the EA fraction revealed similar activity to BHT when the concentration was more than 0.10 mg/mL, which indicates the strong hydroxyl radical scavenging activity.

The EA fraction of different polar fraction derived from crude extracts of the CA6 leaves presented the highest antioxidant activity and flavonoid content under all the tested concentrations. The result was consistent with previous reports on the antioxidant activity and flavonoid content of EA fraction derived from crude extracts of* Dianthus superbus* [28],* Toona sinensis* M. Roem. [29], and* Ruellia tuberosa* [30]. Especially, the CA6 presents the equal antioxidant activity with BHT at a certain concentration and may be a potential resource for development of natural antioxidants.

### 3.4. Isolation and Identification of the Components with Antioxidant Activity

**Figure 2:**
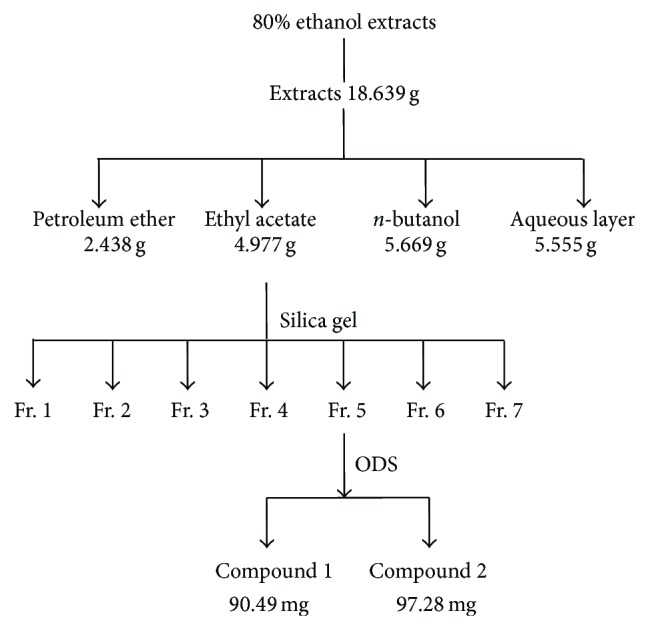
The process of extraction, isolation, and fractionation of bioactive compounds from CA6.

**Figure 3:**
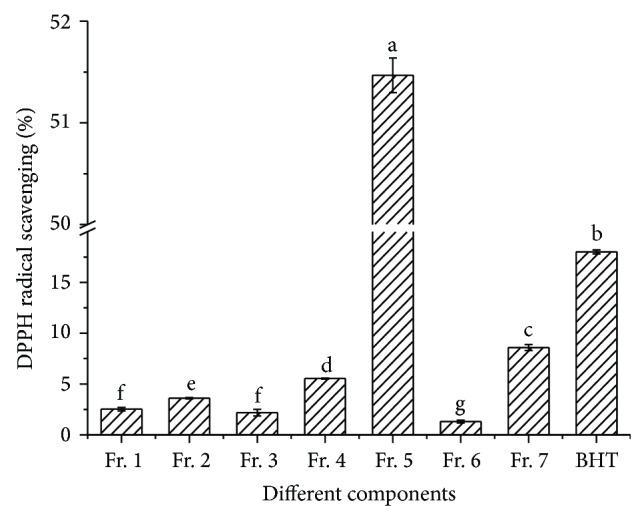
DPPH radical scavenging activity of 7 subfractions.

The EA fraction with the highest antioxidant activity was isolated by silica gel column chromatography, and the eluent was divided into seven fractions (Fractions 1–7) according to the polarity sequence of solvent (Figure 2). Then, the DPPH radical scavenging activity of the seven fractions with the same concentration BHT as the positive control was determined (Figure 3). The DPPH radical scavenging activity of seven fractions and BHT was significantly different (*P* < 0.05) at the concentration of 0.10 mg/mL. Among the seven fractions, the DPPH radical scavenging activity of Fr. 5 was the highest, followed by Fr. 7.

Two compounds** 1 **and** 2** were isolated from Fr. 5 by the ODS column chromatography. The DPPH radical scavenging activity and reducing power of compounds** 1 **and** 2 **at the concentration of 0.10 mg/mL were further investigated. The DPPH radical scavenging activity and reducing power of compound** 1** were 84.04% and 45.58%, respectively, which were higher than those in compound** 2** and BHT at the same concentration.

*Data for* ***1***.* CAS: 128475-17-2,* yellow solid, m.p. 328–330°C; ^1^H NMR (400 MHz, DMSO-*d*_6_)  *δ*: 12.99 (s, 1H, H-5), 9.84 (s, 3H, H-7, 3′, 4′), 7.40–7.44 (m, 2H, H-2′, 6′), 6.88 (d,* J *= 8.0 Hz, 1H, H-5′), 6.68 (s, 1H, H-3), 6.45 (d,* J *= 2.0 Hz, 1H, H-8), 6.19 (d,* J *= 2.0 Hz, 1H, H-6), ^13^C NMR (100 MHz, DMSO-*d*_6_)  *δ*: 182.1 (C-4), 164.6 (C-7), 164.3 (C-2), 161.9 (C-9), 157.7 (C-5), 150.1 (C-4′), 146.1 (C-3′), 121.9 (C-6′), 119.4 (C-1′), 116.4 (C-5′), 113.8 (C-2′), 104.1 (C-3), 103.3 (C-10), 99.2 (C-6), 94.3 (C-8); MS (ESI-TRAP), m/z (%): 285.22 ([M-H]^+^, 100). The data of compound** 1 **was in accordance with the result reported by Pettit et al. [31]. Therefore, compound** 1 **should be luteolin. 

*Data for* ***2***.* CAS: 128475-17-2,* yellow solid, m.p. 345–350°C; ^1^H NMR (400 MHz, DMSO-*d*_6_)  *δ*: 12.98 (s, 1H, H-5), 10.60 (s, 2H, H-7, 4′), 7.92 (d,* J* = 8.8 Hz, 2H, H-2′, 6′), 6.92 (d,* J* = 8.8 Hz, 2H, H-3′, 5′), 6.79 (s, 1H, H-3), 6.49 (d,* J* = 2.0 Hz, 1H, H-8), 6.20 (d,* J* = 2.0 Hz, 1H, H-6); ^13^C NMR (100 MHz, DMSO-*d*_6_)  *δ*: 182.2 (C-4), 164.6 (C-2), 164.1 (C-7), 161.9 (C-4′), 161.6 (C-9), 157.7 (C-5), 128.9 (C-2′, 6′), 121.6 (C-1′), 116.4 (C-3′, 5′), 104.1 (C-10), 103.3 (C-3), 99.2 (C-6), 94.3 (C-8); MS (ESI-TRAP), m/z (%): 269.20 ([M-H]^+^, 100). The data of compound** 2 **was in accordance with the result reported by Ding et al. [32]. Therefore, compound** 2 **should be apigenin.

**Figure 4:**
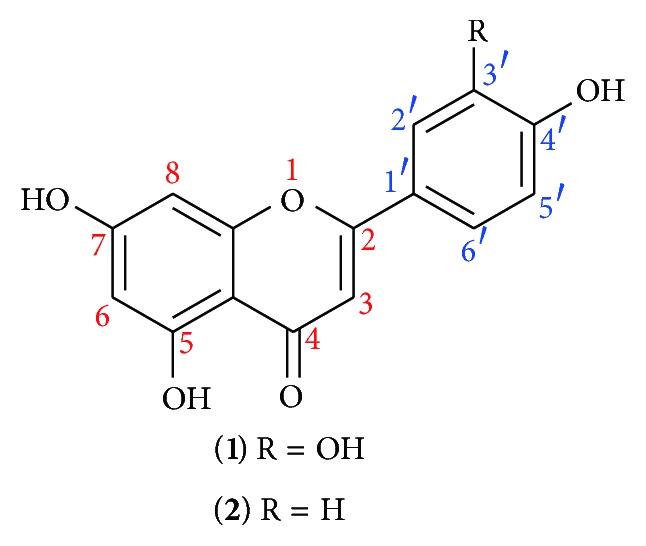
Chemical structures of luteolin (**1**) and apigenin (**2**).

The structures of luteolin (**1**) and apigenin (**2**) (Figure 4) indicate that they are all flavonoid compounds. The difference between in luteolin (**1**) and apigenin (**2**) was only hydroxyl group, but the antioxidant activities of two compounds were significantly different. More hydroxyl groups indicate the stronger antioxidant activities. Besides, both C-3′ and C-4′ of luteolin (**1**) have hydroxyl groups that can form the adjacent two phenolic hydroxyl groups' structure, thus resulting in the higher DPPH radical scavenging activity and reducing power. In conclusion, the number of hydroxyl groups and the structure of adjacent two phenolic hydroxyl groups are the foundation of the highly active flavonoid compounds.

## 4. Conclusions

In this paper, the antioxidant activity of the* C. ovata* G. Don,* C*.* bungei* C. A. Mey.,* C*.* fargesii* Bur., and the 3 clones of* C. bungei* C. A. Mey. was investigated. The results demonstrated that CA6 presented the highest antioxidant activity and the main material with antioxidation ability was medium polar compounds. Moreover, the two flavonoid compounds (luteolin** 1**; apigenin** 2**) from the EA fraction of the CA6 were isolated. This study provided a new plant source for the extraction of luteolin (**1**) and apigenin (**2**). A new way for development and utilization of* Catalpa* plants was also rendered.

## Figures and Tables

**Table 1:** The total flavonoid contents and antioxidant activity of crude extracts from the leaves of *Catalpa* plants.

	Total flavonoids mg/g*·*DW	Reducing power %	DPPH radical scavenging activity IC_50_ mg/mL	Hydroxyl radical scavenging activity IC_50_ mg/mL
*C. ovata *G. Don	29.08 ± 0.157^c^	21.65 ± 0.080^c^	2.35 ± 0.009^a^	0.58 ± 0.011^a^
*C. fargesii* Bur.	32.52 ± 0.102^b^	24.05 ± 0.105^b^	2.01 ± 0.006^b^	0.13 ± 0.001^b^
C. *bungei *C. A. Mey.	40.45 ± 0.143^a^	34.10 ± 0.100^a^	1.73 ± 0.003^c^	0.11 ± 0.001^c^

ANOVA. Different letters within each column indicate the statistical difference according to post hoc comparison (Student Newman Keuls) at *P* < 0.05.

**Table 2:** The total flavonoid contents and antioxidant activity of crude extract from leaves of clones of *C. bungei* C. A. Mey.

Clones	Total flavonoids mg/g*·*DW	Reducing power %	DPPH radical scavenging activity IC_50_ mg/mL	Hydroxyl radical scavenging activity IC_50_ mg/mL
CA2	34.62 ± 0.319^b^	27.49 ± 0.080^b^	1.97 ± 0.004^b^	0.10 ± 0.001^b^
CA5	32.17 ± 0.353^c^	26.58 ± 0.139^c^	2.23 ± 0.008^a^	0.15 ± 0.001^a^
CA6	46.29 ± 0.245^a^	41.61 ± 0.080^a^	0.98 ± 0.008^c^	0.08 ± 0.001^c^

ANOVA. Different letters within each column indicate the statistical difference according to post hoc comparison (Student Newman Keuls) at *P* < 0.05.
